# Multiorgan failure caused by pembrolizumab and axitinib in a woman affected by metastatic clear cell renal cell carcinoma: A case report and literature review

**DOI:** 10.1097/MD.0000000000037606

**Published:** 2024-03-29

**Authors:** Andrea Di Marco, Grazia Artioli, Adolfo Favaretto, Nicolò Cavasin, Umberto Basso

**Affiliations:** a Medical Oncology 1 Unit, Department of Oncology, Istituto Oncologico Veneto IOV IRCCS, Padua, Italy; b Department of Surgery, Oncology and Gastroenterology, University of Padua, Padua, Italy; c Department of Medical Oncology, AULSS 2 Marca Trevigiana, Ca’ Foncello Hospital, Treviso, Italy.

**Keywords:** axitinib, immune checkpoint inhibitors, MOF, pembrolizumab, renal cell carcinoma, tyrosine kinase inhibitors

## Abstract

**Rationale:**

Treatment with a combination of immune checkpoint inhibitors (ICIs) (pembrolizumab or nivolumab) and oral Tyrosine Kinase Inhibitors (TKI) targeting angiogenesis (axitinib, cabozantinib or lenvatinib) has shown benefits in terms of efficacy and survival in metastatic renal cell carcinoma (mRCC), with a favorable toxicity profile. However, some rare and serious treatment-related adverse events can be difficult to manage.

**Patient concerns:**

Here we report the first case of an mRCC patient who, after only 2 administrations of pembrolizumab-axitinib, experienced severe multiorgan failure (MOF) with heart failure, oliguria and acute hepatitis requiring aggressive supportive treatment in intensive care unit.

**Diagnoses:**

A diagnosis of severe MOF induced by pembrolizumab plus axitinib was considered.

**Interventions:**

The patient was treated with dobutamine, levosimendan along with high-dose steroids under continuous cardiologic monitoring.

**Outcomes:**

After treatment, the patient had a full recovery and was discharged from the hospital.

**Lessons:**

We reviewed all the other cases of MOF reported during treatment with combined ICI-TKI in cancer patients in order to summarize incidence, clinical manifestations and management with a specific focus on the need for prompt recognition and aggressive management under multidisciplinary care.

## 1. Introduction

Metastatic Renal Cell Carcinoma (mRCC) has a poor prognosis, with a 5-year relative survival rate of 17% to 18%.^[1]^ The development of agents targeting angiogenesis and modulating the immune system has changed the treatment landscape for patients with mRCC, with impressive response rates exceeding 70%, most of which long lasting.^[2–5]^ Currently, several combinations of an immune checkpoint inhibitor (ICI) plus an oral angiogenesis inhibitor are standard regimens for good, intermediate or poor risk mRCC^[6]^ according to the International Metastatic Consortium Database (IMDC). The most frequently used regimens are the anti-Programmed Cell Death-1 (PD1) antibody pembrolizumab plus axitinib,^[2]^ the anti-PD1 antibody nivolumab plus cabozantinib^[3]^ or pembrolizumab plus lenvatinib.^[4]^

In the open-label, randomized, phase III KEYNOTE-426 trial, a combination of pembrolizumab plus axitinib was compared with sunitinib and showed an improvement in median Progression Free Survival (PFS) (15.1 months in the combination group vs 11.1 months in the sunitinib arm, HR 0.69, 95% CI 0.57–0.84, *P* < .001), as well as in 18-months Overall Survival (OS) (82.3% in the combination group vs 72.1% in the sunitinib arm, HR 0.57, 95% CI 0.38–0.74, *P* < .0001).^[2]^ The benefits of pembrolizumab-axitinib in terms of OS and PFS were observed in all subgroups examined, including all IMDC risk and PD-L1 expression categories. The extended follow-up analysis of the KEYNOTE-426 trial confirmed the previous results (median PFS 15.4 months in the combination group vs 11.1 months, HR 0.71, 95% CI 0.60–0.84, *P* < .0001; 24-months OS 74.4% in the combination group vs 65.5%, HR 0.68, 95% CI 0.55–0.85, *P* = .0003).^[7]^

The most common adverse events related to pembrolizumab are immune-related adverse events (irAEs), which can involve almost any part of the body in the form of pneumonitis, colitis, hepatitis, hypothyroidism/hyperthyroidism, hypophysitis etc., along with more general adverse events (AEs) related to immune activation and systemic inflammation (fatigue, diarrhea, rash, musculoskeletal problems).^[8]^

Axitinib is a selective inhibitor of Vascular Endothelial Growth Factor Receptor 1 (VEGFR-1), VEGFR-2 and VEGFR-3. The most common adverse events include hypertension, diarrhea, fatigue, decreased appetite, nausea, dysphonia, hand-foot syndrome, hypothyroidism and proteinuria.^[9]^

The combination of an antiangiogenetic drug plus an ICI caused higher AEs rates compared to each treatment when administered alone.^[10]^ However, in the KEYNOTE-426 trial the overall frequency of Grade 3 and 4 toxic effects was eventually similar between pembrolizumab plus axitinib and sunitinib alone (75.8% vs 70.6%) as well as rates of toxicity-related discontinuation (10.7% vs 13.9%). Eleven patients (2.6%) in the pembrolizumab-axitinib group died from treatment-related AEs (one patient each from cardiac arrest, myasthenia gravis, myocarditis, necrotizing fasciitis, plasma-cell myeloma, pneumonitis, pulmonary embolism, pulmonary thrombosis, respiratory failure, sudden cardiac death, and death not otherwise specified) compared to 15 with sunitinib (3.5%).^[2]^

Here we report the case of an adult female patient with mRCC who experienced acute multiorgan failure (heart failure, oliguria and acute hepatitis) associated with immune-related thyroiditis after the first 2 administrations of pembrolizumab-axitinib, which required admission to intensive care and aggressive supportive care treatments. A summary of similar cases reported in literature is then provided along with discussion on risk factors, management and outcomes.

## 2. Case presentation

A 57-year-old female with no relevant comorbidities was diagnosed in March 2022 with a large solid left renal mass. A venous thrombus extending to the caval vein along with multiple and bilateral lung metastases were detected by computerized tomography (CT scan). She was classified as intermediate risk due to metastatic onset and anemia according to IMDC criteria. Renal biopsy reported clear cell renal cell carcinoma. The WHO grade and presence of sarcomatoid differentiation could not be determined due to the small size of the tumor sample.

On 6 May, she received the first administration of pembrolizumab 200 mg and started therapy with axitinib, 5 mg twice a day. After 2 weeks, she presented to the Emergency Room complaining from pain in the left hemithorax, extending to the left arm. Electrocardiogram, troponin T and echocardiography results were normal, a contrast-enhancing chest CT scan showed no signs of pulmonary thromboembolism and axitinib was temporarily suspended.

A second cycle of pembrolizumab plus axitinib was precautionally postponed by 1 week to 4 June, and the following day the patient experienced a peak of symptomatic hypertension (160/90 mm Hg) and started Ramipril 5 mg daily with prompt resolution.

On 21 June, the patient was hospitalized for severe worsening of general conditions, nausea, vomiting, mental confusion, oliguria and profuse sweating.

On physical examination the patient showed tachycardia (156 bpm) along with moderate fever (up to 38°C) and moderate swelling of lower limbs. Blood chemistry tests showed increased transaminases (ALT 935 U/L, AST 1659 U/L) with normal bilirubin, mild creatinine elevation (1.05 mg/dL with estimated GFR 45.7 mL/min/1.73 m^2^ according to Cockroft-Gault formula), subclinic hyperthyroidism (TSH 0.01 mUI/L, fT4 4.35 ng/dL), hyperglycemia (11.17 mmol/L), and mild electrolyte imbalance (Na 134 mEq/L, K 5.5 mEq/L). Under suspicion of an acute adrenal insufficiency related to ICI therapy, the dosage of adrenal hormones was tested, with normal results.

The patient started infusion of saline solution plus ceftriaxone, high dose hydrocortisone and B-blocker but her conditions worsened with development of acute heart failure with severe depression of left ventricular ejection fraction (LVEF, 10% at echocardiography) and renal failure with lactic acidosis. A diagnosis of severe multiorgan failure (MOF) induced by pembrolizumab plus axitinib was made.

The patient was therefore transferred to an intensive care unit where she was treated with dobutamine, levosimendan along with high-dose steroids under continuous cardiologic monitoring. The patient then gradually improved and after 1 week returned to the General Medicine ward, from which she was discharged 6 weeks after first admission. Treatment with pembrolizumab-axitinib was not resumed due to the life-threatening toxicity (permanent discontinuation).

By September 2022, the patient had regained fair general conditions. Echocardiography showed recovery of LVEF (50%) with persistence of diastolic dysfunction, with full normalization of liver tests and persistence of mild creatinine elevation (around 1.6 mg/dL with estimated GFR 28.8 mL/min/1.73 m^2^ according to Cockroft-Gault formula). Unfortunately, a CT scan showed progressive disease in lungs, lymphnodes and renal mass. After lengthy discussion with the patient, we agreed that second-line tyrosine kinase inhibitors (TKI) therapy was too dangerous for her and therefore she started treatment with everolimus, 10 mg daily.

This treatment was well tolerated apart for moderate anemia, subsequent echocardiographic assessments showed normal LVEF (57%), liver tests remained normal with mild elevation of creatinine. On January 2023, the new CT scan showed reductions of lung nodules, with stabilization of renal mass and lymphnodes and so everolimus was continued.

Unfortunately, on May 2023 the CT scan revealed progression of lung nodules as well as the appearance of new liver metastases. We discussed at length with the patient the option of starting third-line TKI therapy, but we eventually agreed to deliver only best supportive care. The patient rapidly developed respiratory failure and died on July 7, 2023 due to disease progression.

## 3. Discussion

MOF or multiple organ dysfunction syndrome is defined as “the development of potentially reversible physiologic derangement involving 2 or more organ systems not involved in the disorder that result in Intensive Care Unit (ICU) admission, and arising in the wake of a potentially life threatening physiologic insult.”^[11]^

MOF is characterized by 3 general criteria:

1. organ failure, however determined, must have been present for more than 24 hours;
2. the risk of mortality increases with the number of organs involved;
3. prognosis is related to the duration of the insufficiency itself.

Dysregulated immune response to a critical illness plays a central role in the pathogenesis of MOF: the current view is that organ failure results from loss of homeostasis between systemic inflammation and a counterbalancing anti-inflammatory response.^[12]^

There are several general therapeutic measures for the prevention and resolution of MOF.^[11]^ If the patient is in shock, it must be treated immediately and antibiotics need to be started quickly. If there is a known source of infection, it needs to be removed in order to minimize the inflammation that contributes to the development of MOF. Patients with fluid overload (generally defined as greater than 10% weight increase from baseline) have an increase in MOF-related mortality; therefore, diuretics or dialysis/renal replacement therapy should be used to decrease fluid overload. Lung-protection strategies, such as the use of low tidal volumes of approximately 6 ml/kg should be utilized for patients with acute respiratory failure/acute respiratory distress syndrome requiring mechanical ventilation.

Glycemic control is also important, although the level of hyperglycemia at which treatment should be initiated (i.e., insulin) is not well established. Hyperglycemia is known to deactivate monocytes, increase C-reactive protein and inflammation, and reduce immune function. Early initiation of nutrition is also imperative to maintain healthy commensal gut bacteria and immune system balance. When MOF is refractory to treatment, potential toxicities of concomitant drugs that are known to contribute to MOF should be removed such as cardiotoxic or nephrotoxic drugs.

If an autoimmune mechanism is suspected, such as in immune-related adverse events (irAEs) induced by immune checkpoint inhibitors (ICIs), systemic immunosuppression must be started promptly.^[13]^

Our patient developed a life-threatening form of MOF after only 2 doses of pembrolizumab plus axitinib notwithstanding the absence of previous or concomitant cardiovascular problems. Other cases of MOF following administration of pembrolizumab or axitinib as monotherapy or within combination regimens have already been reported in literature.

Hyun et al reported in 2020 a fatal case of MOF in a 45-year-old woman treated with pembrolizumab for a thymoma refractory to first-line platinum-based chemotherapy; in particular, the patient experienced severe hepatitis, myasthenia gravis and acute fulminant myocarditis (responsible for the fatal outcome).^[14]^

Another case of MOF related to pembrolizumab treatment was reported by Xie et al in 2021 in a 67-year-old Chinese man, treated with pembrolizumab for a large-cell lung neuroendocrine carcinoma. This patient also developed acute hepatitis, myasthenia gravis, and fulminant myocarditis. He recovered from the acute phase of MOF, but he subsequently developed a delayed immune-related pneumonia (about 8 weeks after the end of the immunotherapy treatment), responsive to treatment with methylprednisolone.^[15]^

A retrospective study by Salem et al in 2018 reported the association between ICI treatment and the onset of myocarditis: this AE had a median onset time of 30 days from the start of immunotherapy and in 64% of cases it occurred between the first and the second course of treatment.^[16]^ Moreover, a multicentric study by Mahmood et al showed a 1.14% prevalence of myocarditis in patients treated with ICIs.^[17]^

In 2020, Matsui et al reported the case of a lethal myocarditis and myositis after pembrolizumab treatment in a 69-year-old man affected by advanced upper urinary tract urothelial carcinoma.^[18]^ In this case, the patient experienced myalgia and a slightly elevated creatinine kinase on the day of the second administration of pembrolizumab; 5 days later, the patient was admitted with severe fatigue and abnormal gait and the myocardial biopsy confirmed the diagnosis of myocarditis. This case suggests that myositis precedes myocarditis; therefore, if myositis is suspected, subsequent cardiological assessment is warranted. The authors suggest that a routine follow-up of creatine-phosphokinase and cardiac troponin I, as well as Electrocardiogram monitoring, should be performed to identify early any possible ICI-induced myocarditis and myositis.

The pathophysiology of ICI-related myocarditis is still unclear, but has biologic plausibility: PD-1 plays a role in myocardial immune responses and protects against inflammation and myocyte damage in mice models of T-cell–mediated myocarditis.^[19]^ Genetic deletion of PD-1 in mice leads to cardiomyopathy that is caused by autoantibodies against cardiac troponin I.^[20]^ In 2016, Johnson et al reported 2 cases of fatal myocarditis developed after treatment with combined ipilimumab-nivolumab.^[21]^ In both patients, myositis with rhabdomyolysis and myocarditis with a robust presence of T-cell and macrophage infiltrates was reported. The selective clonal T-cell populations infiltrating the myocardium were identical to those present in tumors and skeletal muscle, which suggests a T-cell-driven drug reaction: T-cells could be targeting an antigen shared by the tumor, skeletal muscle, and the heart, or the same T-cell receptor may be targeting a tumor antigen and a different but homologous muscle antigen. High levels of muscle-specific antigens (desmin and troponin) were observed in tumors from both patients. Further studies are needed to elucidate the causative antigens and molecular mechanisms involved in these events.

With regard to axitinib, heart failure was reported in 1.7% of treated patients with renal cell carcinoma and this finding is similar to that observed for other VEGF inhibitors.^[22]^ There have been several proposed mechanisms for TKI-associated heart failure: one study in mouse model using a tunable transgene encoding a VEGF trap showed that blockade of VEGF leads to decreased myocardial capillary density, induction of hypoxia and hypoxia-inducible genes in the myocardium, and cardiac dysfunction, which is reversible upon removal of the transgene;^[23]^ consistently with this model, many cases of TKI-associated cardiomyopathy are reversible.^[24]^

Axitinib-associated hypertension is frequent and may be associated with cardiovascular events like heart failure, chest pain, dyspnea, arrhythmia and myocarditis. Therefore, continuous monitoring and early medical treatment with antihypertensive agents is recommended for axitinib-associated hypertension.^[13]^

A retrospective observational study by Yokoyama et al in 2021, carried out on a population of patients treated with TKIs directed towards the VEGFR (including axitinib), highlighted a worsening of the diastolic function of the left ventricle as an AE associated with these drugs.^[25]^

In 2020, Tanriverdi et al reported the case of a 70-year-old man with renal cell carcinoma, treated with interferon-alfa in the first line, followed by sunitinib and third line with axitinib.^[26]^ While receiving axitinib, the patient experienced dyspnea, palpitations, and left ventricular dysfunction, with an EF of 35%. This adverse event was successfully treated with diuretics, acetylsalicylic acid, low molecular weight heparin, β-blockers and angiotensin-converting enzyme inhibitors. Once stabilized, the patient started a fourth line of systemic therapy with Nivolumab: during this final treatment, there was no worsening of cardiac function, which gradually recovered.

The exact rate of immune-related cardiac toxicities with the combination of axitinib and ICI is currently not known, and proper monitoring is recommended. Due to the high mortality associated with immune-related myocarditis, aggressive management consist in the prompt initiation of high-dose prednisolone (1–2 mg/kg) or other equivalent corticosteroids. Cardiac monitoring in an intensive care unit should be considered, given the high risk of high-grade conduction delays and ventricular arrhythmias. Additional immunosuppression may be required in severe cases or based on the myocardial biopsy findings, although it is not advisable to delay treatment and wait for the full investigative findings. The decision to treat should be made on a clinical basis.^[13]^

The risk of severe hepatic toxicity is non-negligible for combined pembrolizumab-axitinib as observed in the KEYNOTE-426 trial: grade 3 to 4 alanine aminotransferase (ALT) increase and grade 3 to 4 aspartate aminotransferase (AST) increase were observed respectively in 13.3% and 7% of the patients in the pembrolizumab-axitinib group. Although there were no deaths related to hepatic AEs, the incidence of grade 3 or 4 elevations in liver-enzyme levels in the pembrolizumab–axitinib group was higher than previously observed when each agent was used as monotherapy. Among the 125 patients with ALT ≥ 3x upper limit of normal (ULN), 120/125 (96%) recovered to < 3× ULN following study treatment interruption/discontinuation; 68/120 (57%) received corticosteroids. One hundred patients were rechallenged with one or both study treatment(s): 45/100 (45%) had ALT ≥ 3× ULN recurrence, and all 45 recovered to ALT < 3× ULN following study treatment interruption/discontinuation.^[27]^

No other cases of MOF related to pembrolizumab-axitinib treatment are reported in literature; however, Nakazawa et al in 2022 reported a case of acute heart failure in a woman treated with lenvatinib plus pembrolizumab for a clear cell endometrial carcinoma.^[28]^ The patient was treated with high-dose steroids and made a full recovery, but the treatment with pembrolizumab and lenvatinib was discontinued. Table 1 summarizes published cases of MOF/acute heart failure reported under treatment with pembrolizumab, axitinib or combination regimens.

**Table 1:** List of MOF/acute heart failure cases reported under treatment with pembrolizumab, axitinib or combination regimens.

Report	Sex	Age	Cancer	Oncological treatment	Onset time	Clinical aspects	Supportive treatment	Outcome
Hyun et al, 2020	F	45	Thymoma	Pembrolizumab monotherapy	15 d	Myocarditis, acute hepatitis, myasthenia gravis, altered mental status, cardio-renal syndrome	Extra-Corporeal Membrane Oxygena-tion, high dose i.v. steroids, dialysis	Death
Xie et al, 2021	M	67	Large Cell Neuro-Endocrine Cancer of the Lung	Pembrolizumab plus carboplatin and pemetrexed	15 d	Myocarditis, acute hepatitis, myasthenia gravis, delayed pneumonitis	High dose i.v. steroids, pacemaker implant, antibiotics	Recovery with definitive pace-maker implant
Tanriverdi et al, 2020	M	70	Renal Cell Carcinoma	Axitinib monotherapy	Not reported	Heart failure (Left Ventricular Ejection Fraction 35%)	Diuretics, acetyl-salicylate, low molecular weight heparin, B-blocker, ramipril	Full recovery, started new treatment with Nivolu-mab
Iwasaki et al, 2021	F	70	Hepato-cellular carcinoma	Atezolizu-mab plus Bevacizu-mab	4 d	Myocarditis, heart failure (Left Ventricular Ejection Fraction 40%)	Diuretics, high dose i.v. steroids, high flow oxygen	Full recovery
Nakazawa et al, 2022	F	64	Clear cell endo-metrial cancer	Pembrolizumab plus Lenvatinib	34	Myocarditis, heart failure (Left Ventricular Ejection Fraction 15%)	High dose i.v. steroids	Full recovery
Present article	F	57	Renal Cell Carcinoma	Pembrolizumab plus Axitinib	40 d	Heart Failure (Left Ventricular Ejection Fraction 10%), acute hepatitis, oliguria, altered mental status, thyroiditis	Saline solution, anti-biotics, high dose i.v. steroids, B-blocker, ino-tropics, vaso-dilators	Full recovery

Previous studies showed a correlation between the onset of grade 3 to 4 irAEs and a better response to treatment.^[29]^ In patients with melanoma and renal cancer who received ipilimumab at doses ranging from 3 to 9 mg/kg every 3 weeks, there was a clear association between development of irAEs (mainly gastro-intestinal or endocrine-related) and response to ipilimumab.^[30,31]^ Interestingly, the use of steroids to treat irAEs has not been shown to reduce the benefit of ICI treatment. Teraoka and al. showed a positive association between the onset of early irAEs (which occur 2–6 weeks after the beginning of treatment) and clinical outcome in patients with advanced non-small-cell lung cancer treated with nivolumab.^[32]^

Unfortunately, our patient did not experience a response to pembrolizumab and axitinib treatment despite the onset of an early, life-threatening irAE.

Although the opportunity of ICI rechallenge after interruption due to irAEs can be feasible and effective, the probability of toxicity recurrence is high.^[33,34]^ We discussed with the patient the next line of treatment and we opted for a second-line treatment with everolimus, which has a different mechanism of action and does not cause cardiac toxicity. Unfortunately, the clinical benefit was transient, with an initial partial response and a definitive disease progression after about 8 months of treatment.

We considered the possibility of starting third-line cabozantinib therapy, but, due to the progressive deterioration of the patient’s clinical condition, the high risk of cardiac toxicity and the limited expected clinical benefit, we finally agreed not to start a new treatment and to continue with Best Supportive Care.

## 4. Conclusion

We reported the first case, to our knowledge, of reversible MOF in a patient treated with pembrolizumab and axitinib for mRCC, which was reversible thanks to aggressive supportive treatment. Prompt recognition of MOF is crucial to start aggressive management, usually by a multidisciplinary team including oncologists, cardiologists, nephrologists, anesthesiologists and endocrinologists. Intensive care unit support may be needed.

Rechallenging patients recovering from MOF caused by ICIs and TKIs in combination or by either drug alone is very risky, therefore the use of drugs with different mechanism of action may be reasonable options, such as the m-TOR inhibitor everolimus or, potentially, the new Hypoxia Inducible Factor-2 alfa inhibitor belzutifan.^[35]^

## Author contributions

**Conceptualization:** Andrea Di Marco, Umberto Basso, Grazia Artioli.

**Data curation:** Andrea Di Marco.

**Investigation:** Andrea Di Marco.

**Methodology:** Andrea Di Marco, Grazia Artioli, Umberto Basso.

**Supervision:** Grazia Artioli, Adolfo Favaretto, Umberto Basso.

**Validation:** Umberto Basso.

**Visualization:** Grazia Artioli, Adolfo Favaretto, Nicolò Cavasin, Umberto Basso.

**Writing – original draft:** Andrea Di Marco.

**Writing – review & editing:** Nicolò Cavasin, Umberto Basso.

## References

[1] Surveillance Research Program NCI. SEER*Explorer: an interactive website for SEER cancer statistics; 2023 Apr 19. Available at: https://seer.cancer.gov/statistics-network/explorer/ [access date June 2, 2023].

[2] Rini BI Plimack ER Stus V.; KEYNOTE-426 Investigators. Pembrolizumab plus axitinib versus sunitinib for advanced renal-cell carcinoma. N Engl J Med. 2019;380:1116–27. 30779529 10.1056/NEJMoa1816714

[3] Choueiri TK Powles T Burotto M.; CheckMate 9ER Investigators. Nivolumab plus cabozantinib versus sunitinib for advanced renal-cell carcinoma. N Engl J Med. 2021;384:829–41. 33657295 10.1056/NEJMoa2026982 PMC8436591

[4] Motzer R Alekseev B Rha SY.; CLEAR Trial Investigators. Lenvatinib plus pembrolizumab or everolimus for advanced renal cell carcinoma. N Engl J Med. 2021;384:1289–300. 33616314 10.1056/NEJMoa2035716

[5] Motzer RJ Penkov K Haanen J. Avelumab plus axitinib versus sunitinib for advanced renal-cell carcinoma. N Engl J Med. 2019;380:1103–15. 30779531 10.1056/NEJMoa1816047 PMC6716603

[6] Quhal F Mori K Bruchbacher A. First-line immunotherapy-based combinations for metastatic renal cell carcinoma: a systematic review and network meta-analysis. Eur Urol Oncol. 2021;4:755–65. 33757737 10.1016/j.euo.2021.03.001

[7] Powles T Plimack ER Soulières D. Pembrolizumab plus axitinib versus sunitinib monotherapy as first-line treatment of advanced renal cell carcinoma (KEYNOTE-426): extended follow-up from a randomised, open-label, phase 3 trial. Lancet Oncol. 2020;21:1563–73. 33284113 10.1016/S1470-2045(20)30436-8

[8] Baxi S Yang A Gennarelli RL. Immune-related adverse events for anti-PD-1 and anti-PD-L1 drugs: systematic review and meta-analysis. BMJ. 2018;360:k793. 29540345 10.1136/bmj.k793 PMC5851471

[9] Keating GM. Axitinib: a review in advanced renal cell carcinoma. Drugs. 2015;75:1903–13. 26487541 10.1007/s40265-015-0483-x

[10] Nocera L Karakiewicz PI Wenzel M. Clinical outcomes and adverse events after first-line treatment in metastatic renal cell carcinoma: a systematic review and network meta-analysis. J Urol. 2022;207:16–24. 34546767 10.1097/JU.0000000000002252

[11] Ramírez M. Multiple organ dysfunction syndrome. Curr Probl Pediatr Adolesc Health Care. 2013;43:273–7. 24295608 10.1016/j.cppeds.2013.10.003

[12] Rittirsch D Flierl MA Ward PA. Harmful molecular mechanisms in sepsis. Nat Rev Immunol. 2008;8:776–87. 18802444 10.1038/nri2402 PMC2786961

[13] Grünwald V Voss MH Rini BI. Axitinib plus immune checkpoint inhibitor: evidence- and expert-based consensus recommendation for treatment optimisation and management of related adverse events. Br J Cancer. 2020;123:898–904. 32587360 10.1038/s41416-020-0949-9 PMC7492460

[14] Hyun JW Kim GS Kim SH. Fatal simultaneous multi-organ failure following pembrolizumab treatment for refractory thymoma. Clin Lung Cancer. 2020;21:e74–7. 31718909 10.1016/j.cllc.2019.10.008

[15] Xie X Wang F Qin Y. Case report: fatal multiorgan failure and heterochronous pneumonitis following pembrolizumab treatment in a patient with large-cell neuroendocrine carcinoma of lung. Front Pharmacol. 2020;11:569466. 33584255 10.3389/fphar.2020.569466 PMC7878548

[16] Salem JE Manouchehri A Moey M. Cardiovascular toxicities associated with immune checkpoint inhibitors: an observational, retrospective, pharmacovigilance study. Lancet Oncol. 2018;19:1579–89. 30442497 10.1016/S1470-2045(18)30608-9 PMC6287923

[17] Mahmood SS Fradley MG Cohen JV. Myocarditis in patients treated with immune checkpoint inhibitors. J Am Coll Cardiol. 2018;71:1755–64. 29567210 10.1016/j.jacc.2018.02.037 PMC6196725

[18] Matsui H Kawai T Sato Y. A fatal case of myocarditis following myositis induced by pembrolizumab treatment for metastatic upper urinary tract urothelial carcinoma. Int Heart J. 2020;61:1070–4. 32921673 10.1536/ihj.20-162

[19] Tarrio ML Grabie N xiu BD. PD-1 protects against inflammation and myocyte damage in T cell-mediated myocarditis. J Immunol. 2012;188:4876–84. 22491251 10.4049/jimmunol.1200389 PMC3345066

[20] Okazaki T Tanaka Y Nishio R. Autoantibodies against cardiac troponin I are responsible for dilated cardiomyopathy in PD-1-deficient mice. Nat Med. 2003;9:1477–83. 14595408 10.1038/nm955

[21] Johnson DB Balko JM Compton ML. Fulminant myocarditis with combination immune checkpoint blockade. N Engl J Med. 2016;375:1749–55. 27806233 10.1056/NEJMoa1609214 PMC5247797

[22] Goldman A Bomze D Dankner R. Cardiovascular toxicities of antiangiogenic tyrosine kinase inhibitors: a retrospective, pharmacovigilance study. Target Oncol. 2021;16:471–83. 33970401 10.1007/s11523-021-00817-2

[23] May D Gilon D Djonov V. Transgenic system for conditional induction and rescue of chronic myocardial hibernation provides insights into genomic programs of hibernation. Proc Natl Acad Sci USA. 2008;105:282–7. 18162550 10.1073/pnas.0707778105 PMC2224202

[24] Uraizee I Cheng S Moslehi J. Reversible cardiomyopathy associated with sunitinib and sorafenib. N Engl J Med. 2011;365:1649–50. 22030001 10.1056/NEJMc1108849

[25] Yokoyama H Shioyama W Shintani T. Vascular endothelial growth factor receptor inhibitors impair left ventricular diastolic functions. Int Heart J. 2021;62:1297–304. 34853223 10.1536/ihj.21-307

[26] Tanriverdi O Ates S Sandal KK. Left ventricular dysfunction associated with axitinib and nivolumab experience in an advanced renal cell carcinoma. J Oncol Pharm Pract. 2020;26:1765–8. 32164490 10.1177/1078155220909422

[27] Rini BI Atkins MB Plimack ER. Characterization and management of treatment-emergent hepatic toxicity in patients with advanced renal cell carcinoma receiving first-line pembrolizumab plus axitinib. Results from the KEYNOTE-426 trial. Eur Urol Oncol. 2022;5:225–34. 34244116 10.1016/j.euo.2021.05.007

[28] Nakazawa H Yamaguchi S. Acute heart failure during lenvatinib plus pembrolizumab therapy. Int J Gynecol Cancer. 2022;32:817. 35667674 10.1136/ijgc-2022-003532

[29] Weber JS. Practical management of immune-related adverse events from immune checkpoint protein antibodies for the oncologist. Am Soc Clin Oncol Educ Book. 2012;32:174–7. 10.14694/EdBook_AM.2012.32.79 24451730

[30] Downey SG Klapper JA Smith FO. Prognostic factors related to clinical response in patients with metastatic melanoma treated by CTL-associated antigen-4 blockade. Clin Cancer Res. 2007;13:6681–8. 17982122 10.1158/1078-0432.CCR-07-0187 PMC2147083

[31] Blansfield JA Beck KE Tran K. Cytotoxic T-lymphocyte-associated antigen-4 blockage can induce autoimmune hypophysitis in patients with metastatic melanoma and renal cancer. J Immunother. 2005;28:593–8. 16224277 10.1097/01.cji.0000178913.41256.06 PMC2154350

[32] Teraoka S Fujimoto D Morimoto T. Early immune-related adverse events and association with outcome in advanced non–small cell lung cancer patients treated with nivolumab: a prospective cohort study. J Thorac Oncol. 2017;12:1798–805. 28939128 10.1016/j.jtho.2017.08.022

[33] Bimbatti D Maruzzo M Pierantoni F. Immune checkpoint inhibitors rechallenge in urological tumors: an extensive review of the literature. Crit Rev Oncol Hematol. 2022;170:103579. 35007699 10.1016/j.critrevonc.2022.103579

[34] Abou Alaiwi S Xie W Nassar AH. Safety and efficacy of restarting immune checkpoint inhibitors after clinically significant immune-related adverse events in metastatic renal cell carcinoma. J ImmunoTher Cancer. 2020;8:e000144. 32066646 10.1136/jitc-2019-000144 PMC7057439

[35] Choueiri TK Bauer TM Papadopoulos KP. Inhibition of hypoxia-inducible factor-2α in renal cell carcinoma with belzutifan: a phase 1 trial and biomarker analysis. Nat Med. 2021;27:802–5. 33888901 10.1038/s41591-021-01324-7 PMC9128828

